# Asian women with PCOS have enhanced ovarian reserve and ART outcomes, even at an advanced maternal age: a model for reproductive longevity?

**DOI:** 10.1093/hropen/hoaf062

**Published:** 2025-10-14

**Authors:** Qian Yang, Paula Benny, Jovin Jie Ning Lee, Devi Natalie Nadjaja, Shaili P Sashidharan, Eu-Leong Yong, Mahesh Choolani, Stephen Chew, Ling-Jun Li, Peng Cheang Wong, Zhongwei Huang

**Affiliations:** NUS Bia-Echo Asia Centre of Reproductive Longevity and Equality, Yong Loo Lin School of Medicine, National University of Singapore, Singapore, Singapore; Department of Obstetrics and Gynaecology, Yong Loo Lin School of Medicine, National University of Singapore, Singapore, Singapore; NUS Bia-Echo Asia Centre of Reproductive Longevity and Equality, Yong Loo Lin School of Medicine, National University of Singapore, Singapore, Singapore; Department of Obstetrics and Gynaecology, Yong Loo Lin School of Medicine, National University of Singapore, Singapore, Singapore; NUS Bia-Echo Asia Centre of Reproductive Longevity and Equality, Yong Loo Lin School of Medicine, National University of Singapore, Singapore, Singapore; Department of Obstetrics and Gynaecology, Yong Loo Lin School of Medicine, National University of Singapore, Singapore, Singapore; NUS Bia-Echo Asia Centre of Reproductive Longevity and Equality, Yong Loo Lin School of Medicine, National University of Singapore, Singapore, Singapore; Department of Obstetrics and Gynaecology, Yong Loo Lin School of Medicine, National University of Singapore, Singapore, Singapore; NUS Bia-Echo Asia Centre of Reproductive Longevity and Equality, Yong Loo Lin School of Medicine, National University of Singapore, Singapore, Singapore; Department of Obstetrics and Gynaecology, Yong Loo Lin School of Medicine, National University of Singapore, Singapore, Singapore; Department of Obstetrics and Gynaecology, Yong Loo Lin School of Medicine, National University of Singapore, Singapore, Singapore; Department of Obstetrics and Gynaecology, National University Centre for Women and Children (NUWoC), National University Health System, Singapore, Singapore; Department of Obstetrics and Gynaecology, Yong Loo Lin School of Medicine, National University of Singapore, Singapore, Singapore; Department of Obstetrics and Gynaecology, National University Centre for Women and Children (NUWoC), National University Health System, Singapore, Singapore; Department of Obstetrics and Gynaecology, Yong Loo Lin School of Medicine, National University of Singapore, Singapore, Singapore; Department of Obstetrics and Gynaecology, National University Centre for Women and Children (NUWoC), National University Health System, Singapore, Singapore; NUS Bia-Echo Asia Centre of Reproductive Longevity and Equality, Yong Loo Lin School of Medicine, National University of Singapore, Singapore, Singapore; Department of Obstetrics and Gynaecology, Yong Loo Lin School of Medicine, National University of Singapore, Singapore, Singapore; Global Centre for Asian Women Health, Yong Loo Lin School of Medicine, National University of Singapore, Singapore, Singapore; Department of Obstetrics and Gynaecology, Yong Loo Lin School of Medicine, National University of Singapore, Singapore, Singapore; Department of Obstetrics and Gynaecology, National University Centre for Women and Children (NUWoC), National University Health System, Singapore, Singapore; NUS Bia-Echo Asia Centre of Reproductive Longevity and Equality, Yong Loo Lin School of Medicine, National University of Singapore, Singapore, Singapore; Department of Obstetrics and Gynaecology, Yong Loo Lin School of Medicine, National University of Singapore, Singapore, Singapore; Department of Obstetrics and Gynaecology, National University Centre for Women and Children (NUWoC), National University Health System, Singapore, Singapore

**Keywords:** polycystic ovary syndrome, anti-Müllerian hormone, ART, human reproduction, reproductive longevity, Asian women

## Abstract

**STUDY QUESTION:**

Does polycystic ovary syndrome (PCOS) represent a human model for reproductive longevity?

**SUMMARY ANSWER:**

Asian women with PCOS have enhanced ovarian reserve and ART outcomes, even at an advanced maternal age.

**WHAT IS KNOWN ALREADY:**

PCOS afflicts 4–20% of women at reproductive age and is associated with anovulation, hyperandrogenism, and polycystic ovarian morphology. However, there remains a paucity in research into the PCOS-related reproductive outcomes in Asian women following ART. This study addresses a critical gap in elucidating the Asian PCOS phenotype and reproductive longevity associated with PCOS, within the context of a multi-ethnic Asian population.

**STUDY DESIGN, SIZE, DURATION:**

A retrospective observational cohort with a total of 3092 women from a tertiary-care centre in Singapore was analysed in this study.

**PARTICIPANTS/MATERIALS, SETTING, METHODS:**

PCOS was diagnosed according to the 2003 Rotterdam Criteria. After exclusions, 1249 women were grouped into the PCOS (n = 212) or normo-ovulatory (n = 1037) groups. Clinical demographics, ART protocols, reproductive outcomes, and hormone levels were evaluated in the study. Modified Poisson Regression analyses were used to compare the ART outcomes between PCOS and normo-ovulatory groups.

**MAIN RESULTS AND THE ROLE OF CHANCE:**

Women with PCOS exhibit elevated levels of anti-Müllerian hormone (AMH) in comparison to normo-ovulatory women. While AMH levels typically decreased with age, the decline was observed to be significantly slower in women with PCOS when compared to their normo-ovulatory counterparts. Even after the age of 36 years, women with PCOS maintained relatively higher AMH levels (PCOS vs normo-ovulatory: 44.4 vs 19.3 pmol/l). The cumulative pregnancy rate following one ovarian stimulation cycle of ART decreased with age in normo-ovulatory women after 30 years old: 46.0% for ages 31–35 and 28.6% for ages 36 and older (*P* < 0.001). Conversely, for women with PCOS following ART, cumulative pregnancy rates remained stable in advanced maternal age, namely 56.7% for ages 31–35 and 55.9% for ages 36 and older. Compared with the normo-ovulatory group, the adjusted relative risk (aRR) of cumulative pregnancy rates in the PCOS group was significantly higher for women aged 36 years and older undergoing ART (aRR: 1.78; 95% CI: 1.24–2.54), especially for those undergoing IVF (2.01; 1.40–3.14).

**LIMITATIONS, REASONS FOR CAUTION:**

This retrospective study included only Asian women, and hence this may not be applicable to other non-Asian populations.

**WIDER IMPLICATIONS OF THE FINDINGS:**

Our findings provide strong support for our hypothesis that women with PCOS may exhibit an extended reproductive life span and could attain successful pregnancy outcomes through ART, even at advanced maternal ages. These results demonstrate that women with PCOS are likely to have an enhanced reproductive lifespan and warrant further prospective longitudinal studies to unravel the mechanisms underlying the reproductive longevity observed in women with PCOS.

**STUDY FUNDING/COMPETING INTEREST(S):**

This work was funded in part by the NUS Bia-Echo Asia Centre for Reproductive Longevity and Equality. All authors declare that no competing interests exist.

**TRIAL REGISTRATION NUMBER:**

NA.

WHAT DOES THIS MEAN FOR PATIENTS? Polycystic ovary syndrome (PCOS) affects between 4 and 20% of women of reproductive age. Based on the Rotterdam criteria, women with PCOS can have any two out of three conditions: irregular menstrual cycles, hyperandrogenism, and polycystic ovaries, all of which can adversely affect fertility. In our study of Asian women, it was observed that women with PCOS did not experience an age-associated reduction of anti-Müllerian hormone levels (a marker of the number of oocytes present in the ovary) as compared to women without PCOS, meaning that women with PCOS tend to have a higher ovarian reserve even at an older maternal age (≥36 years old). In addition, older women with PCOS who underwent assisted reproduction had a higher number of oocytes retrieved and higher cumulative pregnancy rates as compared to women of the same age without PCOS. These findings suggest that women with PCOS may have an extended reproductive lifespan, which warrants further studies into the biological mechanisms governing reproductive longevity.

## Introduction

Polycystic ovary syndrome (PCOS), defined by the Rotterdam criteria established in 2003, involves the presence of at least two out of the following three symptomatic manifestations: oligo/amenorrhoea, hyperandrogenism (HA), and the presence of polycystic ovarian morphology (PCOM) (Rotterdam ESHRE/ASRM-Sponsored PCOS Consensus Workshop Group, 2004). Recently, anti-Müllerian hormone (AMH), a member of the transforming growth factor-β superfamily, which is secreted by growing ovarian antral follicles, has emerged as a biomarker to predict ovarian aging and reproductive lifespan (Sowers *et al.*, 2008; van Disseldorp *et al.*, 2008; Dólleman *et al.*, 2013; Iino *et al.*, 2013; Depmann *et al.*, 2016; Minooee *et al.*, 2018) as well as a diagnostic criterion (Deswal *et al.*, 2020) for PCOS.

PCOS affects a substantial proportion of women of reproductive age, ranging from 4 to 20%, and stands as one of the most prevalent causes of female infertility. It also contributes to other long-term health risks and medical complications such as diabetes mellitus and psychological issues (Li *et al.*, 2013; Deswal *et al.*, 2020). However, recent studies suggest that women with PCOS experience a delayed onset of ovarian aging and changes to menstrual cycles as they age, with women in this group reaching menopause approximately 2 years later than those with regular ovulation (Elting *et al.*, 2000; Tehrani *et al.*, 2010). While some research indicates better reproductive outcomes in older women with PCOS (Siristatidis *et al.*, 2015; Tannus *et al.*, 2018), conflicting findings exist, with certain studies showing no significant differences in live birth or clinical pregnancy rates between PCOS and non-PCOS groups (Zhang *et al.*, 2023). However, these studies had small sample sizes and did not account for heterogeneity due to ethnic variations.

It has previously been reported that ethnic differences exist in women undergoing ART in the USA (SART; Society for Assisted Reproductive Technology; Wellons *et al.*, 2012), with Asians having a lower clinical pregnancy rate (OR: 0.71; 95% CI: 0.64–0.80) and decreased live birth rate (OR: 0.69; 95% CI: 0.61–0.77), as compared to Caucasian sub-fertile women undergoing IVF (Purcell *et al.*, 2007; Fujimoto *et al.*, 2010). However, the general lack of data in multi-ethnic Asian populations and disaccord of PCOS diagnosis criteria further compound this issue. In addition, the trends in delayed childbearing and increasing use of ART in fertility assistance necessitate a new look into PCOS-related reproductive outcomes following ART.

Therefore, we conducted a large retrospective study to investigate whether women with PCOS in a multi-ethnic Asian cohort in Singapore exhibit different pregnancy outcomes compared to normo-ovulatory women who underwent ART, especially at advanced maternal ages. We hypothesize that Asian women with PCOS experience different reproductive outcomes following ART, potentially due to the unique reproductive lifespan and potential in women with PCOS.

## Materials and methods

### Study design and participants

**Figure 1.**
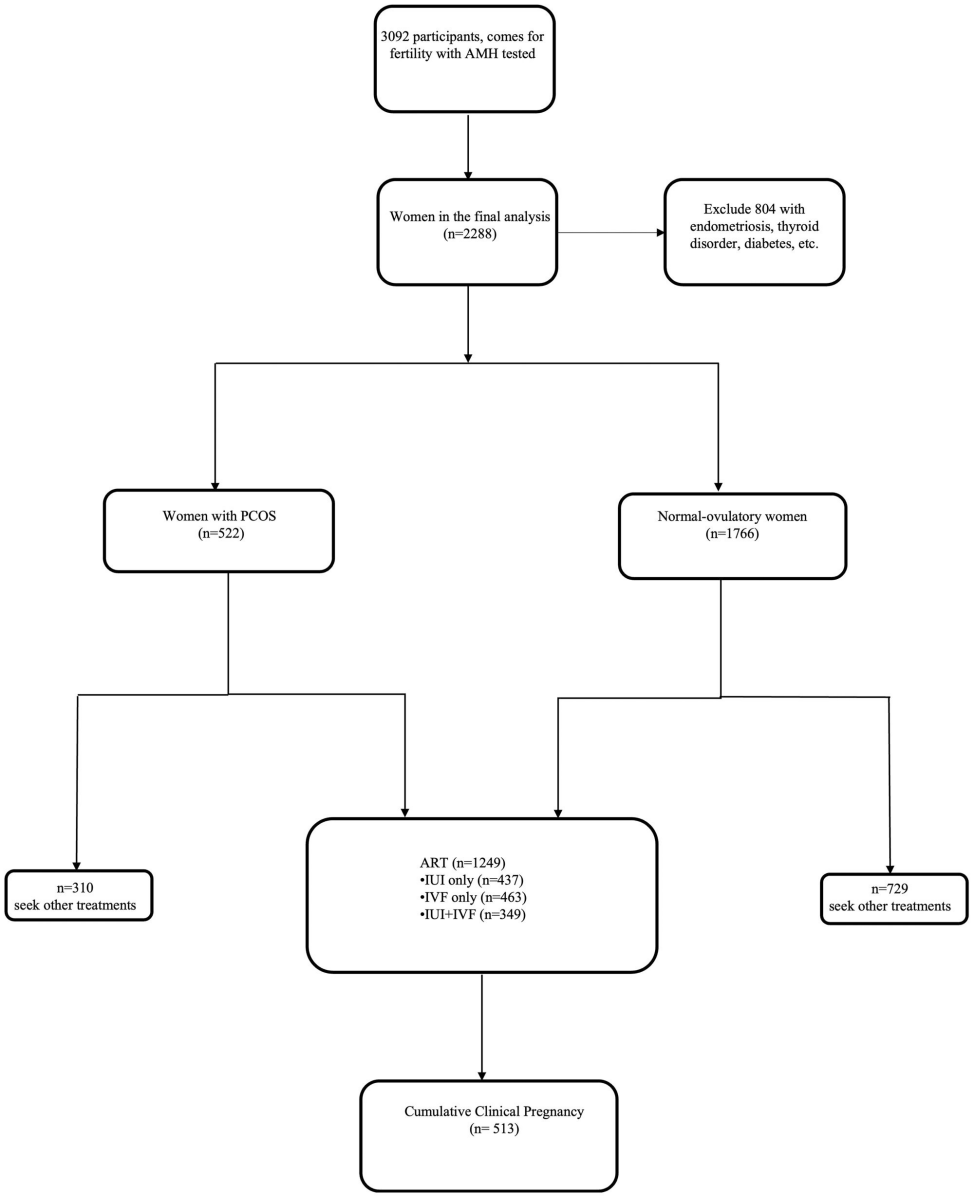
**Study participants with inclusion and exclusion criteria.** PCOS, polycystic ovary syndrome.

A retrospective cohort study was conducted at the National University Hospital, Department of Obstetrics and Gynaecology, in Singapore. Women who sought fertility assistance between 2016 and 2022 and who were naïve to any IVF treatments were reviewed for potential inclusion. Approval for the study was obtained from the local ethics committee board (DSRB 2020/01033). Women without AMH test results were excluded from the analysis. Additionally, individuals with endocrine disorders (such as thyroid diseases, Cushing’s syndrome, androgen-secreting tumours), endometriosis, breast cancer, diabetes mellitus, or a history of hormonal contraception (within 3 months) were also excluded from the study cohort (Fig. 1).

PCOS was diagnosed by a physician based on the 2003 Rotterdam criteria, with the presence of at least two out of the following three symptomatic manifestations: oligo/amenorrhoea (absence of menstruation for ≥35 days), HA (clinically indicated by an mF-G score of ≥6, with or without acne, and/or androgenic alopecia, biochemically demonstrated by androstenedione levels exceeding 10.8 nmol/l or total testosterone levels surpassing 2.81 nmol/l), and the presence of PCOM characterized by an antral follicle count exceeding 12 and ovarian volume exceeding 10 ml (Rotterdam ESHRE/ASRM-Sponsored PCOS Consensus Workshop Group, 2004). All participants in the control group exhibited regular menstrual cycles, had normal androgen levels, did not present with polycystic ovaries upon ultrasound examination, and had no other existing medical conditions but needed fertility care due to indications such as tubal factor in the female, male factor infertility, and unexplained couple infertility with failed IUI cycles. Clinical demographics were also collected from study participants. Healthy BMI was defined as 18.5–22.9, following BMI cut-offs for Asians. Blood samples were sent to NUH Referral Labs for quantification of AMH, LH, FSH, and testosterone levels. AMH levels were measured using an AMH Plus chemiluminescent immunoassay and Roche Cobas E801 analytical unit (Roche Diagnostics, Risch-Rotkreuz, Switzerland). LH, FSH, testosterone levels were measured using chemiluminescent immunoassays on the Beckman Coulter DXI system (Beckman Coulter, CA, USA).

### IUI, IVF (ICSI), and embryo transfer protocol

For IUI procedures, the attending physician will prescribe ovulation induction agents such as clomiphene/letrozole taken during Days 2–6 of menses and then monitor either ovary to detect if there is the presence of a dominant ovarian follicle developing up to 17–18 mm. Once the dominant ovarian follicle achieves 17–18 mm in dimension, a hCG trigger (Ovidrel^®^ 250 μg/0.5 ml, Merck Pte Ltd, Singapore) will be self-administered by the patient 36 h prior to IUI. This is to mimic the LH surge to initiate maturation of the oocyte and ovulation. Sperm samples were collected in the morning of the procedure, processed as described below, and inseminated into the womb cavity.

For IUI procedures, semen samples were collected in the morning before the procedure. Initial assessments on the liquefaction, volume, viscosity, concentration, motility, and morphology were done prior to treatment. The semen samples were then processed through either the direct or indirect swim-up method by layering the semen sample with media followed by subsequent incubation. For the direct swim-up method, the semen sample was directly layered with the media and incubated at 37°C at 45° inclination. The harvesting of highly motile sperm was done by collecting the topmost part of the media following the incubation step. The harvested sample was washed through centrifugation at 250*g* for 5 min before being transferred into the final tube. For indirect swim-up, the semen sample was first washed with media at a 1:1 sample-to-media ratio at 250*g* for 5 min. The resuspended samples were then layered with media at 1:1 ratio and incubated at 37°C at 45° inclination. Following incubation, the highly motile sperm were collected from the topmost part of the media and transferred into the final tube. A final assessment on the volume, concentration, motility, and morphology was also carried out from the final tube prior to the insemination procedure.

According to the parameters established by the World Health Organization on the severity of decline in sperm progressive motility, concentration, and morphology, patients were categorized into three subgroups. The severe group included men who were oligozoospermic with a concentration of <5 × 10^6^ sperm/ml, and/or asthenozoospermic with a progressive motility of <10%, and/or teratozoospermic with <1% normal morphology. The mild to moderate group included oligozoospermic men whose concentrations ranged from 5 to <15 × 10^6^ sperm/ml, and/or asthenozoospermic men with progressive motility ranging from 10% to <32%, and/or men with normal morphology ranging from 1% to <4%. The normozoospermic group included those with a concentration of 15 × 10^6^ sperm/ml or higher, progressive motility of 32% or higher, and normal morphology of 4% or higher (Yu *et al.*, 2022).

For IVF cycles, almost all followed the GnRH antagonist protocol for ovarian stimulation. Follicle growth was monitored by ultrasonography and sex hormone levels at regular intervals. GnRH agonist or hCG trigger or both (dual trigger) were administered to trigger final oocyte maturation when at least 4 follicles measured ≥17 mm. Oocyte retrieval was performed 34–36 h after trigger injection. For the majority of the patients, only one embryo at the cleavage stage or blastocyst stage was transferred, with remaining embryos being vitrified and frozen. Luteal phase support by subcutaneous hCG may be started for fresh embryo transfer during IVF cycles that utilize the GnRH agonist trigger only. Routinely, oral progesterone would be started on the day of oocyte retrieval, and vaginal progesterone would be commenced the day after oocyte retrieval.

For frozen embryo transfers, endometrial preparation was performed. ICSI was routinely performed for all cases whereby the male partner had abnormal semen parameters. Oral oestradiol in graduated dosages started from 2 mg/day for 4 days, then increased to 4 mg/day for 4 days, and up to 6 mg/day until the day of embryo transfer. When the endometrial thickness reached ≥8 mm and was designated as the day of ovulation, vaginal progesterone (Crinone 8%/90 mg vaginal progesterone, Merck Pte Ltd., Singapore) would be commenced a day later. Embryo thawing and embryo transfer were performed on Day 4 of progesterone initiation for cleavage stage embryos and Day 5 for blastocysts. Luteal phase support was continued until 12 weeks of gestation once pregnancy was confirmed.

### Outcomes

Clinical pregnancy (primary outcome) was defined as a gestational sac with foetal heartbeat detected by ultrasound post-embryo transfer 28–30 days, coincident with positive β-hCG. Live birth was defined as the delivery of a live-born infant after 24 weeks of gestation. Pregnancies that resulted in a miscarriage were also recorded. Cumulative clinical pregnancy refers to pregnancies from IVF-naïve women after one ovarian stimulation cycle, which may have led to multiple embryo transfer cycles. Preimplantation genetic testing for aneuploidy (PGT-A) was not conducted routinely during the study period due to the nation’s legislature, which did not allow the clinical routine use of PGT-A.

### Statistical analysis

Statistical analysis was performed using R version 4.2.2 (R Core Team, Vienna, Austria). Descriptive statistics were generated to facilitate inter-group comparisons. Continuous variables were checked for normality, and means were presented with SD, or medians and ranges, as deemed appropriate. Normally distributed variables were compared using Student’s *t*-test and the ANOVA test, while the Mann–Whitney *U*-test and Kruskal–Wallis test were applied to skewed distributed variables. Categorical variables were compared using Pearson’s chi-squared test. Correlations between variables were evaluated with Spearman’s correlation coefficients. Skewed variables were logarithmically transformed before proceeding to downstream analysis. A cubic spline model was used to derive the optimal cut-offs of AHM for reproductive age (Barrio *et al.*, 2013) in both PCOS and normo-ovulatory groups. Modified Poisson Regression analysis was performed to compare the ART outcomes between PCOS and normo-ovulatory groups.

## Results

After excluding 804 individuals with endocrine disorders (such as thyroid diseases, Cushing’s syndrome, androgen-secreting tumours), endometriosis, diabetes mellitus, or a history of hormonal contraception (within 3 months), a total of 1249 women were included in the analysis (n = 212 PCOS, n = 1037 normo-ovulatory women). Subject demographics and clinical, endocrine, and ultrasound characteristics are presented in Table 1. Age, ethnicity, BMI, hormone levels (AMH, FSH, LH, and testosterone), and smoking status were all found to be significantly different between PCOS and normo-ovulatory groups, as shown in Table 1 and Supplementary Table S1 (*P* < 0.05). AMH levels in PCOS versus normo-ovulatory women were 44.4 vs 19.3 pmol/l. In our unique multi-ethnic Singaporean cohort, a stratification of normo-ovulatory women into their ethnic groups showed that Chinese women consistently trended towards higher AMH levels, even when stratified according to their respective age groups (Supplementary Table S2). In addition, ethnic-specific differences in PCOS were observed, with PCOS being most prevalent in Malays (34.6%), followed by Indians (33.1%), other Asians (23.6%), and lastly Chinese (16.5%), as shown in Supplementary Table S3. Upon analysis of the clinical aspects associated with PCOS, the majority of multi-ethnic women diagnosed with PCOS, using the Rotterdam criteria, had oligomenorrhoea/ovulation disorder and polycystic ovaries (80.3%): 10.7% of women had all three characteristics of PCOS (HA, oligomenorrhoea/ovulation disorder, and polycystic ovaries), 5.7% had HA and polycystic ovaries, while 2.9% had HA and oligomenorrhoea/ovulation disorders, as summarized in Supplementary Table S4.

**Table 1.** Comparisons of baseline characteristics of patients who underwent ART (n = 1249).

Characteristics	Overall (n = 1249)	PCOS (n = 212)	Control (n = 1037)	*P* value
	Mean (SD), median (IQR), or n (%)	Mean (SD), median (IQR), or n (%)	Mean (SD), median (IQR), or n (%)	
**Age, years**	33.9 (3.79)	31.7 (3.68)	34.4 (3.66)	<0.001
**Race/ethnicity**				
Chinese	775 (62.0%)	102 (48.1%)	673 (64.9%)	<0.001
Malay	181 (14.5%)	52 (24.5%)	129 (12.4%)	
Indian	158 (12.6%)	38 (17.9%)	120 (11.6%)	
Others	135 (10.9%)	20 (9.4%)	115 (11.1%)	
**BMI, kg/m^2^**	23.9 (4.71)	26.2 (5.59)	23.4 (4.35)	<0.001
**Smoking, yes**	74 (5.9%)	17 (8.0%)	57 (5.5%)	0.23
**Nulliparous, yes**	1075 (86.1%)	187 (88.2%)	888 (85.6%)	0.37
**Male infertility, yes**	1135 (90.9%)	196 (92.5%)	939 (90.5%)	0.50
**Male infertility,**				
Normal–mild	76 (6.1%)	8 (3.8%)	68 (6.5%)	0.31
Mild–moderate	638 (51.1%)	108 (50.9%)	530 (51.1%)	
Severe	483 (38.7%)	85 (40.1%)	398 (38.4%)	
**ART**				
IUI only	437 (35.0%)	95 (44.8%)	342 (33.0%)	0.003
IVF only	463 (37.1%)	62 (29.2%)	401 (38.7%)	
IUI and IVF	349 (27.9%)	55 (25.9%)	294 (28.4%)	
**Cumulative clinical pregnancy, yes**	513 (41.1%)	112 (52.8%)	401 (38.7%)	<0.001
IUI only	119 (27.2%)	36 (37.9%)	83 (24.3%)	0.012
IVF only	205 (44.3%)	43 (69.4%)	162 (40.4%)	<0.001
IUI and IVF	188 (53.9%)	33 (60.0%)	155 (52.7%)	0.41
**Cumulative clinical pregnancy (ART), yes**				
≤30 years old	110 (44.5%)	38 (46.9%)	72 (43.4%)	0.697
31–35 years old	266 (47.8%)	55 (56.7%)	211 (46.0%)	0.070
≥36 years old	137 (30.7%)	19 (55.9%)	118 (28.6%)	0.002
AMH, pmol/l*	22.2 (24.40)	44.4 (30.7)	19.3 (18.5)	<0.001
FSH, IU/l*	7.00 (2.40)	6.20 (2.10)	7.20 (2.40)	<0.001
LH, IU/l*	4.30 (2.60)	6.20 (6.80)	4.10 (2.20)	<0.001
Testosterone, nmol/l*	1.28 (0.820)	1.76 (0.920)	1.17 (0.745)	<0.001

Normal distributed variables were compared using Student’s *t*-test and ANOVA test. Mann–Whitney *U*-test and Kruskal–Wallis test were applied to skewed distributed variables. Categorical variables were compared using Pearson’s chi-squared test.

* Skewed distribution.

AMH, anti-Müllerian hormone; PCOS, polycystic ovary syndrome.

**Figure 2.**
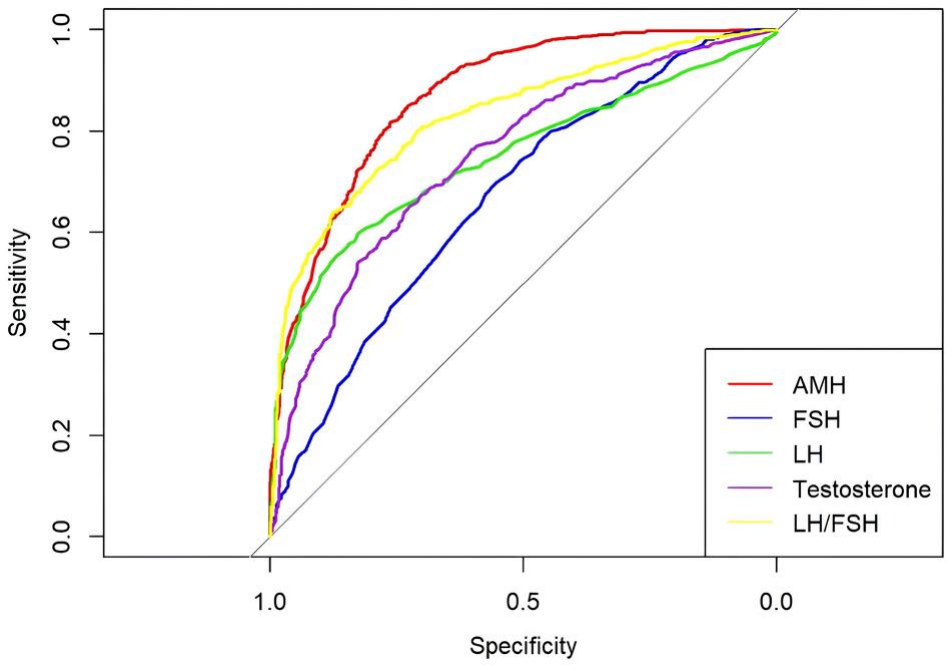
**Receiver operating characteristic (ROC) analysis showing the performance of various hormones as potential PCOS biomarkers.** AMH, anti-Müllerian hormone.

**Figure 3.**
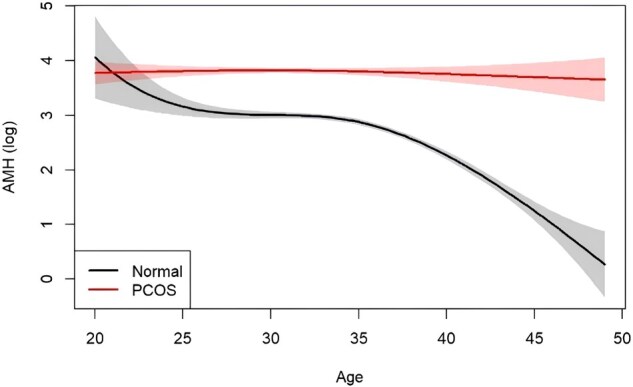
**Cubic spline model plot of the relationship between age and AMH.** AMH, anti-Müllerian hormone; PCOS, polycystic ovary syndrome.

Receiver operating characteristic (ROC) analysis of female sex hormones (AMH, FSH, LH, testosterone, and LH/FSH) showed that AMH had the best performance in predicting PCOS compared with other biomarkers (Fig. 2). We observed that in this cohort, AMH levels decreased with age, as expected (Fig. 3). Noteworthy was the comparatively attenuated rate of decline in AMH levels observed in women with PCOS as opposed to non-PCOS women. Furthermore, within the normo-ovulatory women subset, a marked reduction in AMH levels was observed once a woman was over 35 years old. Plotting of a cubic spline model of the relationship between age and AMH (Fig. 3) identified three optimal cut-off points for age, suggesting that age groups could be subsequently classified to ≤30, 31∼35, and ≥36 years. This classification justifies cut-offs which align with current practice (Stolwijk *et al.*, 2000) and facilitates the derivation of true reproductive age while maintaining an approximately balanced sample size across the different age groups.

A detailed analysis of reproductive outcomes was conducted for women undergoing ART. It was observed that women with PCOS exhibited significantly higher cumulative pregnancy rates compared to the control group, as shown in Table 1 (52.8% vs 38.7%, respectively; *P* < 0.001). This trend remained consistent when ART was stratified into two categories: IUI only and IVF only. The higher pregnancy rates for the PCOS group persisted when the cohort was divided into three age groups (≤30, 31–35, and ≥36 years old), as shown in Table 1 and Supplementary Table S5. The cumulative pregnancy rate following one ovarian stimulation cycle of ART decreased with age in normo-ovulatory women after 30 years old: 46.0% for ages 31–35 and 28.6% for ages 36 and older (*P* < 0.001). Conversely, for women with PCOS following ART, cumulative pregnancy rates remained stable in advanced maternal age, namely 56.7% for ages 31–35 and 55.9% for ages 36 and older.

**Figure 4.**
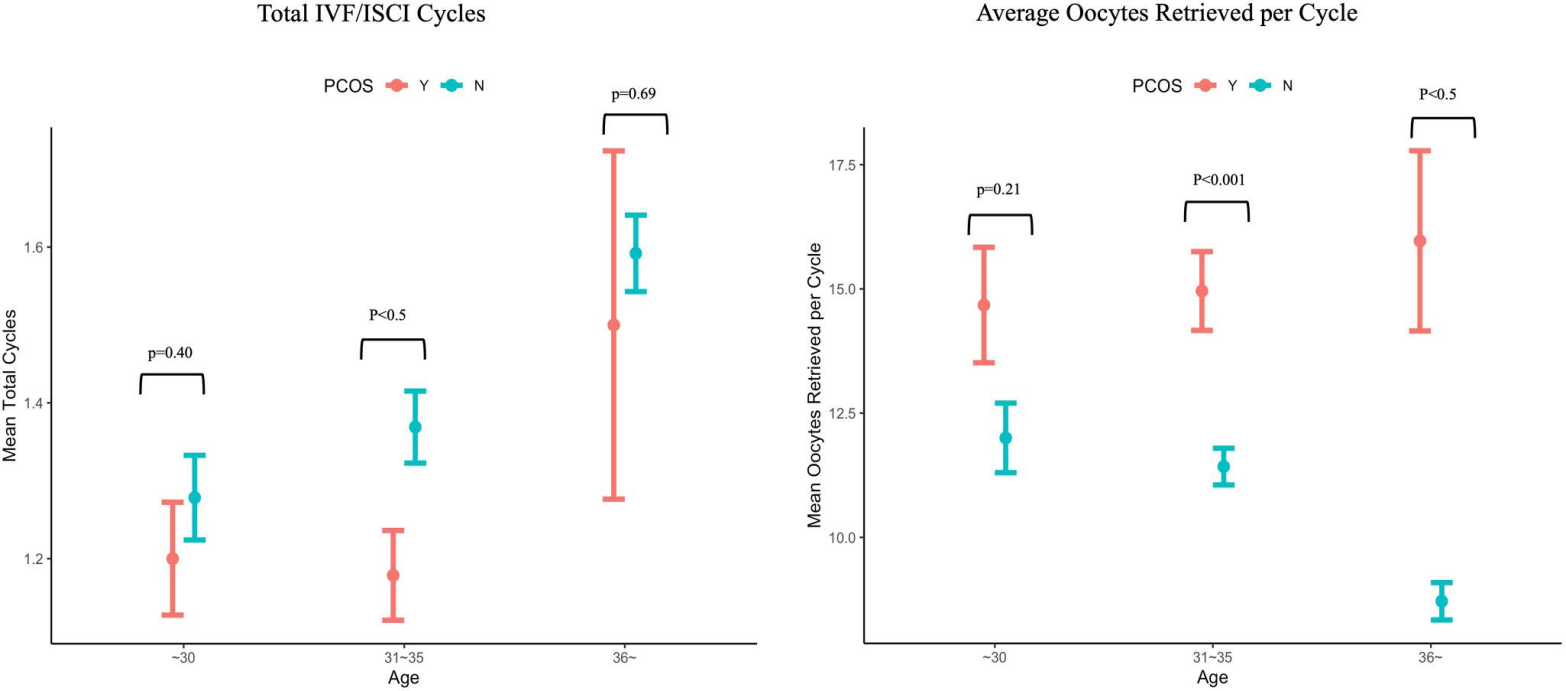
**Comparisons of total numbers of cycles and average number of oocytes retrieved per cycle between PCOS and normo-ovulatory women who underwent IVF/ICSI.** PCOS, polycystic ovary syndrome.

Upon analysis of only the women who underwent IVF, the PCOS group was found to have a higher average number of oocytes retrieved per cycle (Fig. 4) and higher pregnancy rates compared to the control group (Supplementary Table S6), even at advanced maternal ages (≥36 years old). Of note, the number of IVF cycles was controlled for to ensure that the cumulative pregnancy rate was not simply due to a patient undergoing more IVF cycles.

As a measure of oocyte quality, the number of fertilized oocytes and usable blastocysts (Day 5 + Day 6 blastocysts) per oocyte retrieval was also analysed (Supplementary Table S7). It was observed that more fertilized oocytes (8.77 vs 5.76, *P* < 0.001) and more usable blastocysts were obtained (4.35 vs 2.35, *P* < 0.001) in women with PCOS as compared to normo-ovulatory women. Even after controlling for age, the significant difference remained. In addition, women with PCOS had more fertilized oocytes/oocyte harvested (0.554 vs 0.496, *P* = 0.11) and more usable blastocysts/oocyte harvested (0.271 vs 0.162, *P* = 0.002) as compared to normo-ovulatory women. After controlling for age, the number of usable blastocysts/oocytes harvested was significantly higher in women with PCOS as compared to the control group. Similarly, women with PCOS had significantly more fertilized oocytes per oocyte harvested after adjusting for age (*P* = 0.04).

It was also observed that the correlation between oestradiol (E2) levels and the total number of oocytes retrieved was positive overall with a significant correlation coefficient, Spearman ρ = 0.4, *P* < 0.0001 (Supplementary Fig. S1). In addition, the E2:oocyte ratio (EOR; oestradiol levels measured just before oocyte retrieval, per number of oocytes retrieved) was higher in women with PCOS as compared to normo-ovulatory women (1560 vs 1480), as shown in Supplementary Table S8. Every effort was made to retrieve E2 levels on the day of the ovulation trigger; however, some patients had their last E2 levels measured between 2 and 4 days before oocyte retrieval. E2 levels measured more than 5 days before oocyte retrieval were not included. EOR remained relatively constant even as women with PCOS aged. Average E2 levels (measured just before oocyte retrieval) were higher in women with PCOS as compared to normo-ovulatory women (20 400 vs 14 300 pmol/l), and this trend was observed in all age groups (≤30, 31–35, and ≥36 years old). Even at an advanced maternal age (≥36 years old), E2 levels remained high in women with PCOS as compared to normo-ovulatory women who undergo an age-associated decline in E2 levels. In our cohort, E2 levels (measured just before oocyte retrieval) were highest among Malays (19,200 pmol/l), followed by Chinese (15 000 pmol/l), other Asians (14 600 pmol/l) then Indians (13 100 pmol/l), *P* = 0.002, though the number of oocytes retrieved was not significantly different between the ethnicities (*P* = 0.079), as shown in Supplementary Table S9. Every effort was made to retrieve E2 levels on the day of trigger for oocyte retrieval; however, some patients had their last E2 levels measured between 2 and 4 days before oocyte retrieval. E2 measured more than 5 days before oocyte retrieval was not included. The EOR was highest among other Asians (1730 pmol/l) followed by Malays (1610 pmol/l), Chinese (1450 pmol/l), then Indians (1370 pmol/l), *P* = 0.003.

**Figure 5.**
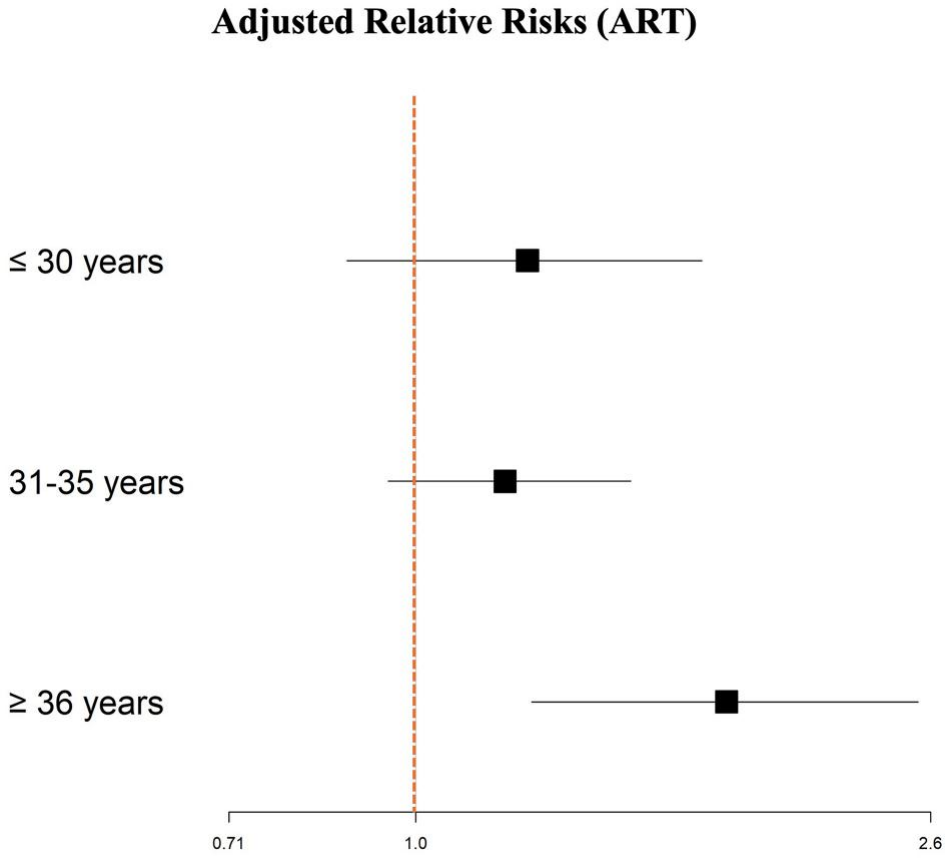
**Comparisons of adjusted cumulative clinical pregnancy relative risks of patients between PCOS and normo-ovulatory women who underwent ART including IUI and IVF/ICSI.** Bonferroni correction was applied for multiple comparisons.

**Figure 6.**
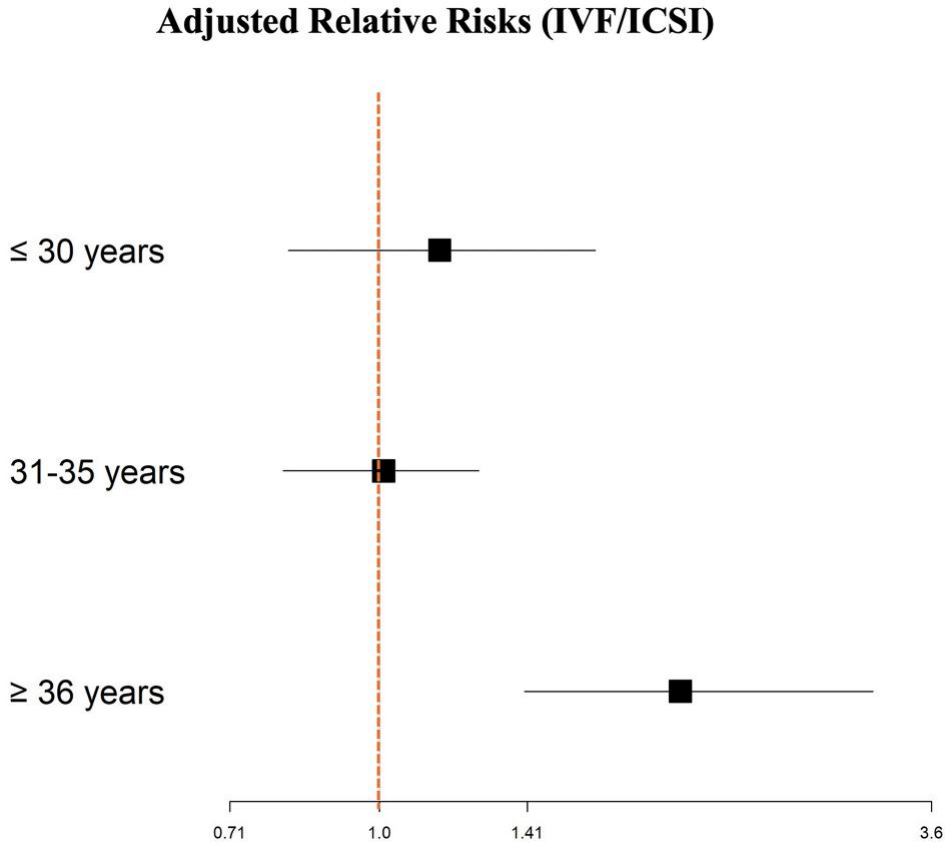
**Comparisons of adjusted cumulative clinical pregnancy relative risks between PCOS and normo-ovulatory women who underwent only IVF/ICSI.** Bonferroni correction was applied for multiple comparisons.

While the pregnancy rate decreased with age in the control group, this trend was not observed in the PCOS group, as illustrated in Supplementary Table S6. Of note, the number of IVF cycles with frozen embryo transfers was controlled for to ensure that the cumulative pregnancy rate was not simply due to a patient undergoing more IVF cycles. Additionally, after adjusting for BMI, ethnicity, severity of male infertility, and smoking status, the relative risk of (cumulative) pregnancy in the PCOS group was significantly higher for women ≥36 years old undergoing ART as compared to those in the normo-ovulatory group (adjusted relative risk (aRR): 1.78; 95% CI: 1.24–2.54; Table 2 and Fig. 5) and especially for those undergoing IVF (aRR: 2.01; 95% CI: 1.40–3.14; Table 3 and Fig. 6). However, in terms of live birth rates, although they were higher in the PCOS group compared with the normo-ovulatory group for women 36 years and older, the difference was not statistically significant.

**Table 2.** Modified Poisson Regression between PCOS and normo-ovulatory groups with pregnancy outcomes among women who underwent ART (n = 1249).

Maternal age, years	Cumulative pregnancy rate		Cumulative live birth rate	
PCOS vs normo-ovulatory (reference)	Relative risk (95% CI)	Adjusted relative risk (95% CI)	Relative risk (95% CI)	Adjusted relative risk (95% CI)
**Overall (n = 1245)**				
	1.36 (1.17, 1.58)	1.22 (1.02, 1.45)	1.06 (0.95, 1.17)	1.06 (0.95, 1.19)
**Stratified by age groups**				
≤30 (n = 247)	1.08 (0.81, 1.44)	1.23 (0.88, 1.70)	0.96 (0.79, 1.16)	1.05 (0.85, 1.31)
31∼35 (n = 556)	1.23 (1.01, 1.51)	1.18 (0.95, 1.49)	1.04 (0.91, 1.19)	1.07 (0.94, 1.21)
≥36 (n = 442)	1.93 (1.38, 2.70)	1.78 (1.24 2.54)	1.22 (0.94, 1.57)	1.31 (0.94, 1.79)

Modified Poisson Regression analysis was performed to compare the ART outcomes between PCOS and normo-ovulatory groups. Models were adjusted for age at baseline, BMI, ethnicity, smoking status and severity of male infertility.

PCOS, polycystic ovary syndrome.

**Table 3.** Modified Poisson Regression between PCOS and normo-ovulatory groups with pregnancy outcomes among women who underwent IVF/ICSI (n = 812).

Maternal age, years	Cumulative pregnancy rate		Cumulative live birth rate	
PCOS vs normo-ovulatory (reference)	Relative risk (95% CI)	Adjusted relative risk (95% CI)	Relative risk (95% CI)	Adjusted relative risk (95% CI)
**Overall (n = 809)**				
	1.42 (1.21, 1.66)	1.12 (0.92, 1.35)	1.04 (0.92, 1.17)	1.01 (0.87, 1.17)
**Stratified by age groups**				
≤30 (n = 138)	1.04 (0.79, 1.38)	1.15 (0.81, 1.65)	0.89 (0.70, 1.13)	0.99 (0.73, 1.33)
31∼35 (n = 350)	1.09 (0.87, 1.35)	1.01 (0.80, 1.26)	1.11 (0.97, 1.27)	1.09 (0.95, 1.25)
≥36 (n = 321)	2.51 (1.79, 3.52)	2.01 (1.40, 3.14)	1.06 (0.74, 1.51)	1.10 (0.72, 1.70)

Modified Poisson Regression analysis was performed to compare the ART outcomes between PCOS and normo-ovulatory groups. Models were adjusted for age at baseline, BMI, ethnicity, smoking status, severity of male infertility, and total IVF cycles.

PCOS, polycystic ovary syndrome.

## Discussion

This study comprised a unique multi-ethnic PCOS cohort from Singapore (normo-ovulatory, n = 1037; PCOS, n = 212), which significantly showed better reproductive outcomes, pregnancy rates, and a higher number of retrieved oocytes for women with PCOS undergoing ART compared to controls, even at older maternal ages (≥36 years old). Second, this study evaluated two indicators of oocyte quality (EOR and blastocyst-to-oocyte ratio) and the differences observed in women with PCOS as compared with normo-ovulatory women of the same age. Third, this study discusses real-world data on the clinical aspects of PCOS and prevalence, unique to a multi-ethnic Asian population.

The PCOS diagnosis criteria have been changing over the decades (1990 NIH, 2003 Rotterdam, 2006 AE-PCOS Society, 2012 NIH/International Guidelines 2018, 2023 International Evidence-based Guidelines), with country-specific adoption making the diagnosis of PCOS exceedingly challenging (Teede *et al.*, 2023). Furthermore, in-depth analyses of reproductive outcomes afflicting women with PCOS are lacking, and the handful of studies that investigated the association of PCOS and reproductive outcomes have reported inconsistent findings. Magnifying this issue is the paucity of research in the Asian demographic, despite the high PCOS prevalence rates. With an increasing proportion of women opting to postpone childbearing to an older age, coupled with the growing acceptance of ART across the globe, it is crucial to bridge the knowledge gaps on the reproductive outcomes in women with PCOS.

Our findings shed light on the reproductive potential of older women with PCOS and the clinical features associated with Asian women. Asian women with PCOS (≥36 years old) had significantly improved reproductive outcomes in terms of higher cumulative pregnancy rates, higher oocyte yield per cycle (Liu *et al.*, 2020; Muharam *et al.*, 2022), and higher oocyte quality (as measured by usable blastocyst-to-oocyte retrieved ratio and fertilized oocytes to oocyte retrieved ratio) as compared to normo-ovulatory women of the same age group.

The EOR was also found to be relatively constant in our study, even as women with PCOS aged (EOR = 1560), in contrast with that in normo-ovulatory women. In comparison with a similar study focused on EOR (Vaughan *et al.*, 2016), the authors showed that clinical pregnancy rates were highest in patients with 250 < EOR < 750 and declined as this ratio increases with EOR > 1000, associated with older maternal age (>36 years old). In addition, EOR was in the range of 1370–1730 for multi-ethnic Asian populations in Singapore. In comparison with a landmark study (Iko *et al.*, 2018) analysing oestradiol/follicle ratio in Asians and Caucasians (and converting pg/ml to pmol/l for E2), we observed that Asian populations in Singapore had much higher E2 levels (13 100–19 200 pmol/l) as compared to American Asians (10 687 pmol/l) (and American Asians had higher E2 compared to American Caucasians, 9538 pmol/l). In addition, Iko *et al.* reported a range of E2/follicle ratios of 539–592 pmol/l for select Asian populations (Japanese, Filipino, Korean, Chinese/Taiwanese, Indian/Pakistani, and Vietnamese/Thai).

Findings from our study (a multi-ethnic Asian cohort, including women with PCOS) provide real-world data that complements studies such as those above (though the American cohort does not exclusively include women with PCOS) to evaluate the potential of using EOR as an indicator of oocyte quality for women undergoing IVF. Some studies have shown that high oestradiol levels tend to be associated with potentially higher number of oocytes retrieved, but very high EOR could indicate poor oocyte quality (Li *et al.*, 2021).

In addition, PCOS prevalence in our cohort resonated with the largest study analysing racial and ethnic differences in infertile women (with and without PCOS) undergoing IVF in the USA (SART CORS; Society for Assisted Reproductive Technology Clinic Outcome Reporting System database) (Mora Calderon *et al.*, 2025), where the authors reported that out of 256 018 patient records obtained, 16.9% met the definition of PCOS, which is comparable to the PCOS prevalence reported in our study (22.8%). Within the PCOS group in SART CORS, there were 71.5% Caucasians, 16% Asians, 7.3% Hispanic, and 5.1% African-American. Our study, comprising of multi-ethnic Asians in Singapore, complements other studies with representations of Asian data. Our findings demonstrate real-world data on multi-ethnic Asian women undergoing IVF (PCOS and normo-ovulatory).

The upper age limit within the ≥36-year-old group (n = 84, 6.7%) was 42 years. We posit that this is attributed to the return of regular ovulatory cycles with age in women with PCOS. Furthermore, it is postulated that polycystic ovaries have a higher initial population of primordial follicles and reduced atresia due to the increased ovarian volume (Webber *et al.*, 2003). However, the significant difference observed in cumulative pregnancy rates did not extend to live birth rates in our cohort, potentially due to our modest sample size after age stratification, as well as due to the limitation of a retrospective study design where all live births were not documented, as women could opt to deliver other than in the hospital where they had their IVF treatments (52% of live birth data points were not complete), and the fact that PGT-A was not routinely carried out during the study period (whereby PGT-A was not allowed in Singapore). In addition, the age limit (45 years old) and cap on the number of ART cycles were only lifted by local health regulations in early 2020. Therefore, the impact of advanced maternal age may be an important determinant of live birth outcomes despite having more oocytes. However, it is interesting to note that in this study population, miscarriage rates were observed to be lower in the ≥36-year-old group (21.4% PCOS vs 23.8% normo-ovulatory group), although the difference was not statistically significant.

Elevated AMH levels are a hallmark of PCOS, and these observations were replicated in our cohort (Mahajan and Kaur, 2019). Declines in AMH levels and ovarian reserve were markedly slower as women with PCOS age, and this was confirmed in our cohort of Asian women, as has been documented in a non-Asian cohort (Alsamarai *et al*, 2009). Higher circulating levels of AMH in older women with PCOS, coupled with a higher number of retrieved oocytes (during IVF), suggest differences in the follicular content of their ovaries, and prior research has shown that it is not due to the increase in primordial follicle recruitment nor changes in atresia, rather it is due to the slow primary follicle growth and stockpiling of classic primary follicles occurring in the setting of PCOS (Maciel *et al.*, 2004). In addition, granulosa and theca cells at the primary follicle stage (where theca cells are recruited to the primordial follicle that consists of pre-granulosa cells) are steroid- and gonadotropin-independent (Huang and Wells, 2010). This may suggest the importance of AMH and its actions on maintaining the primordial follicle and primary follicle pool (Sonigo *et al*, 2019). Asian women with PCOS also had lower FSH and higher testosterone levels, although within the stipulated range of the testosterone assay, which is unique in Asian women with PCOS (Huang and Yong, 2016). Features such as age, BMI, ethnicity, and smoking status significantly impacted AMH levels. These results demonstrate that women with PCOS are likely to have enhanced reproductive lifespans and warrant further prospective longitudinal studies to unravel the mechanisms underlying the reproductive longevity observed in women with PCOS.

### Clinical implications

Investigating the potential extension of reproductive lifespan in women with PCOS holds paramount importance, as it can contribute valuable insights to their unique reproductive trajectories. Furthermore, assessing whether women with PCOS, particularly those at advanced maternal age, could achieve improved pregnancy outcomes through ART is of significant clinical relevance, particularly as the average childbearing age continues to increase worldwide, more women start to seek fertility assistance through ART, and women with PCOS tend to reach menopause later than their normo-ovulatory counterparts (Minooee *et al.*, 2018) and have sustained fertility (Mellembakken *et al.*, 2011). Finally, this is one of a handful of studies to describe AMH levels within a multi-ethnic Singaporean cohort, with Chinese women having consistently higher AMH levels as compared to Indian and Malay women. As with similar cohort studies, raising the awareness of ethnic-specific ovarian reserve would equip women and medical practitioners with important information for a timely diagnosis (Forslund *et al.*, 2025) to guide family planning (Melado *et al.*, 2021). With global secular trends of couples wanting to delay childbearing, elucidating the molecular mechanisms governing reproductive potential could help women to achieve their reproductive aspirations and may provide novel insights into enhancing reproductive lifespan for healthy longevity.

### Strengths and limitations

A major strength of this study is the direct evidence presented showing improved reproductive outcomes in terms of pregnancy rates in women with PCOS, even at advanced maternal ages (≥36 years old). The study goes one step further and shows that women with PCOS have higher oocyte retrieval rates per cycle during ART as compared to normo-ovulatory women, even at advanced maternal ages (≥36 years old). Furthermore, this study includes several ethnic groups residing in Singapore, which is a unique study strength, as Asian populations tend to be under-represented in large cohort studies.

One of the limitations of this study was that the PCOS subjects were younger than the control group, likely because women with PCOS tend to seek medical consultation at a younger age due to issues with irregular menstrual cycles and trying to conceive. To address this, we used a cubic spline model to identify thresholds and justify cut-off points in line with current practice, which divide the cohort into three age groups, potentially reflecting true reproductive age. Another limitation includes the retrospective study design, and as the study time was during the COVID-19 pandemic, not all live birth outcomes were documented, and several women opted to deliver in other hospitals, resulting in missing data on live birth outcomes.

Our study describes the Asian PCOS-related reproductive outcomes following ART in Singapore, with trends reported on ethnicity-associated, ovarian-specific AMH variability. This new knowledge will be valuable, facilitating healthcare professionals to counsel women with PCOS accurately to personalize and optimize their fertility treatments. Future directions resulting from this study are the initiation of a large longitudinal Asian PCOS cohort study to validate our findings on longitudinal reproductive outcomes and to elucidate the molecular mechanisms underlying reproductive longevity in women with PCOS.

## Conclusion

Importantly, this study demonstrates the potential in studying PCOS as a human model for reproductive longevity as evidenced by the enhanced reproductive outcomes. We have demonstrated that Asian women with PCOS benefit from a longer ovarian lifespan due to a high ovarian reserve persisting as they age, thus increasing the likelihood of conceiving even at advanced maternal ages. This retrospective study fills the gap in understanding the reproductive outcomes of Asian women with ovarian conditions such as PCOS and contributes to the understanding of the impact of ethnicities, genetics, and ovarian reserves to personalize and optimize reproductive potentials.

## Supplementary Material

hoaf062_Supplementary_Data

## Data Availability

The data underlying this article will be shared on reasonable request to the corresponding author.
