# Supplementary material for: Asian women with PCOS have enhanced ovarian reserve and ART outcomes, even at an advanced maternal age: a model for reproductive longevity?
Source: Hum Reprod Open. 2025 Oct 14;2025(4):hoaf062. doi: 10.1093/hropen/hoaf062 (PMC12587411; doi:10.1093/hropen/hoaf062)
Supplement: hoaf062_Supplementary_Data [file hoaf062_supplementary_data.zip › Supplementary_Figure_S1_EO.docx]

***
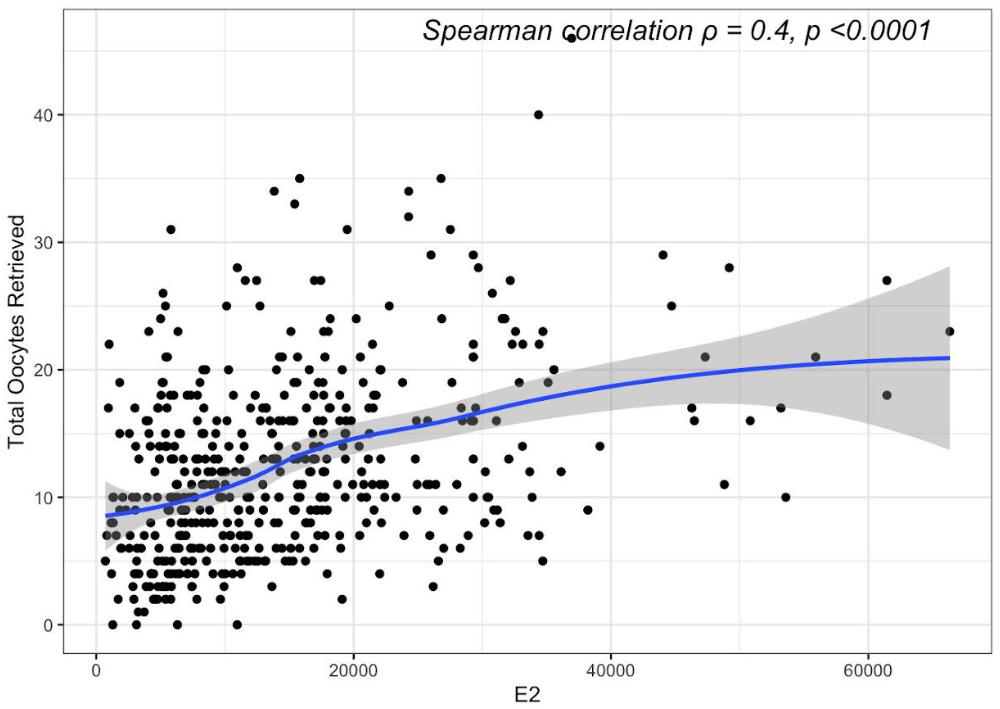
***

**Supplementary Figure S1 Scatter plot showing the association between E2 levels and total oocytes retrieved, showing an overall positive correlation.**
