# Supplementary material for: Asian women with PCOS have enhanced ovarian reserve and ART outcomes, even at an advanced maternal age: a model for reproductive longevity?
Source: Hum Reprod Open. 2025 Oct 14;2025(4):hoaf062. doi: 10.1093/hropen/hoaf062 (PMC12587411; doi:10.1093/hropen/hoaf062)
Supplement: hoaf062_Supplementary_Data [file hoaf062_supplementary_data.zip › HRO-25-0206-R2-SuppTablesS1toS9_EO.docx]

**Supplementary Table S1. Comparisons of baseline characteristics of PCOS and normo-ovulatory women (n=2288)**

| **Characteristics** | **Overall**  **(n=2288)** | **PCOS**  **(n=522)** | **Control**  **(n=1766)** | ***P* value** |
| --- | --- | --- | --- | --- |
|  | Mean (SD) or Median (IQR) or  n (%) | Mean (SD) or Median (IQR) or  n (%) | Mean (SD) or Median (IQR) or  n (%) |  |
| **Age, years** | 33.4 (4.11) | 31.0 (3.77) | 34.1 (3.94) | <0.001 |
| **Race/ethnicity** |  |  |  |  |
| Chinese | 1270 (55.5%) | 209 (40.0%) | 1061 (60.1%) | <0.001 |
| Malay | 350 (15.3%) | 121 (23.2%) | 229 (13.0%) |  |
| Indian | 363 (15.9%) | 120 (23.0%) | 243 (13.7%) |  |
| Others | 305 (13.3%) | 72 (13.8%) | 233 (13.2%) |  |
| **BMI, kg/m^2^** | 24.6 (5.13) | 27.3 (5.85) | 23.8 (4.59) | <0.001 |
| Underweight, <18.5 | 108 (4.7%) | 18 (3.4%) | 90 (5.1%) |  |
| Normal, 18.5-22.9 | 900 (39.3%) | 126 (24.1%) | 774 (43.8%) |  |
| Overweight, 23-24.9 | 344 (15.1%) | 57 (10.9%) | 287 (16.3%) |  |
| Obese, ≥25 | 826 (36.1%) | 306 (58.6%) | 520 (29.4%) |  |
| **Smoking, Yes** | 150 (6.6%) | 49 (9.4%) | 101 (5.7%) | 0.002 |
| **ART, Yes** | 1249 (54.7%) | 212 (40.6%) | 1037 (58.7%) | <0.001 |
| **AMH, pmol/L*** | 22.7 (25.6) | 44.4 (32.0) | 18.5 (18.6) | <0.001 |
| **FSH, IU/L*** | 7.00 (2.38) | 6.10 (1.93) | 7.20 (2.40) | <0.001 |
| **LH, IU/L*** | 4.40 (3.00) | 6.90 (6.95) | 4.10 (2.30) | <0.001 |
| **Testosterone, nmol/L*** | 1.30 (0.870) | 1.76 (0.990) | 1.18 (0.790) | <0.001 |

Normal distributed variables were compared using Student’s t-test and ANOVA test. Mann-Whitney U test and Kruskal–Wallis test were applied to skewed distributed variables. Categorical variables were compared using Pearson’s chi-squared test. (AMH, anti-Müllerian hormone; ART, assisted reproductive technology; BMI, body mass index; FSH, follicle stimulating hormone; LH, luteinizing hormone; PCOS, polycystic ovary syndrome) * Skewed distribution

**Supplementary Table S2: Comparisons of baseline hormone levels of normal-ovulatory women across different ethnicities**

| **Age, years** | **≤30** | | | **31~35** | | | **≥ 36** | | |
| --- | --- | --- | --- | --- | --- | --- | --- | --- | --- |
| Race | Chinese | Malay | Indian | Chinese | Malay | Indian | Chinese | Malay | Indian |
| AMH, pmol/L* | 25.3 (20.6) | 23.8 (17.8) | 17.2 (19.0) | 22.0 (18.8) | 16.4 (15.6) | 19.0 (17.6) | 14.6 (16.0) | 11.0 (13.6) | 11.5 (13.8) |
| *P* value | 0.03 | | | 0.004 | | | 0.003 | | |
| FSH, IU/L* | 6.95 (2.30) | 7.15 (2.43) | 7.00 (2.00) | 7.10 (2.10) | 7.10 (1.90) | 7.20 (1.80) | 7.50 (2.80) | 7.90 (3.20) | 7.70 (2.70) |
| *P* value | 0.99 | | | 0.99 | | | 0.37 | | |
| LH, IU/L* | 4.20 (2.10) | 3.95 (2.40) | 4.65 (3.05) | 4.20 (2.40) | 3.90 (1.78) | 4.70 (2.90) | 3.90 (2.10) | 4.40 (2.50) | 4.00 (2.10) |
| *P* value | 0.70 | | | 0.10 | | | 0.04 | | |
| Testosterone, nmol/L* | 1.29 (0.69) | 1.38 (0.77) | 1.19 (1.13) | 1.18 (0.78) | 1.22 (0.82) | 1.19 (0.73) | 1.12 (0.72) | 1.06 (0.89) | 0.95 (0.98) |
| *P* value | 0.99 | | | 0.99 | | | 0.99 | | |

ANCOVA model for *P* value, accounting for age. AMH, anti-Müllerian hormone; FSH, follicle stimulating hormone; LH,luteinizing hormone *Bonferroni correction was applied for multiple comparisons.

**Supplementary Table S3: Incidence of PCOS in multi-ethnic Asian ethnicities**

|  | **Chinese** | **Malay** | **Indian** | **Others** | **Overall** |
| --- | --- | --- | --- | --- | --- |
|  | **(n=1270)** | **(n=350)** | **(n=363)** | **(n=305)** | **(n=2288)** |
| **PCOS** | 209 (16.5%) | 121 (34.6%) | 120 (33.1%) | 72 (23.6%) | 522 (22.8%) |
| **Control** | 1061 (83.5%) | 229 (65.4%) | 243 (66.9%) | 233 (76.4%) | 1766 (77.2%) |

PCOS, polycystic ovary syndrome.

**Supplementary Table S4: Clinical aspects of PCOS in a multi-ethnic cohort, characterized by the Rotterdam criteria**

|  |  | **Overall (n=522)** |
| --- | --- | --- |
| **HO** | **N** | 507 (97.1%) |
|  | **Y** | 15 (2.9%) |
| **HP** | **N** | 492 (94.3%) |
|  | **Y** | 30 (5.7%) |
| **OP** | **N** | 103 (19.7%) |
|  | **Y** | 419 (80.3%) |
| **HOP** | **N** | 466 (89.3%) |
|  | **Y** | 56 (10.7%) |

PCOS, polycystic ovary syndrome; Y, yes; N, no; H, hyperandrogenism; O, oligomenorrhea/ovulation disorder; P, polycystic ovaries

**Supplementary Table S5. Comparisons of pregnancy outcomes of patients who underwent ART (n=1249)**

|  | **≤30 years** | | **31~35 years** | | **≥36years** | |
| --- | --- | --- | --- | --- | --- | --- |
| **Characteristics** | PCOS  (n=81) | Control  (n=166) | PCOS  (n=97) | Control  (n=459) | PCOS  (n=34) | Control  (n=412) |
|  | Mean (SD)  or  n (%) | Mean (SD)  or  n (%) | Mean (SD)  or  n (%) | Mean (SD)  or  n (%) | Mean (SD)  or  n (%) | Mean (SD)  or  n (%) |
| **Age, years** | 28.1 (1.92) | 28.7 (1.60) | 32.7 (1.38) | 33.1 (1.36) | 37.6 (1.58) | 38.0 (1.69) |
| **BMI, kg/m^2^** | 28.1 (5.42) | 24.4 (4.92) | 25.3 (5.42) | 22.8 (3.72) | 24.3 (5.28) | 23.7 (4.67) |
| **Male infertility, Yes** | 76 (93.8%) | 157 (94.6%) | 89 (91.8%) | 413 (90.0%) | 31 (91.2%) | 369 (89.6%) |
| Normal–mild | 3 (3.7%) | 7 (4.2%) | 4 (4.1%) | 36 (7.8%) | 1 (2.9%) | 25 (6.1%) |
| Mild–moderate | 41 (50.6%) | 76 (45.8%) | 49 (50.5%) | 251 (54.7%) | 18 (52.9%) | 203 (49.3%) |
| Severe | 35 (43.2%) | 81 (48.8%) | 38 (39.2%) | 157 (34.2%) | 12 (35.3%) | 160 (38.8%) |
| **Cumulative clinical pregnancy, Yes** | 38 (46.9%) | 72 (43.4%) | 55 (56.7%) | 211 (46.0%) | 19 (55.9%) | 118 (28.6%) |
| **Live birth, Yes** | 32/38 (84.2%) | 65/72 (90.3%) | 42/55 (76.4%) | 172/211 (81.5%) | 14/19 (73.7%) | 79/118 (66.9%) |
| **Age at delivery** | 31.4 (1.76) | 31.3 (2.12) | 34.8 (1.87) | 35.0 (1.78) | 39.7 (1.63) | 39.3 (1.76) |

Cumulative clinical pregnancy refers to pregnancies from IVF naïve women after one ovarian stimulation cycle, which may have led to multiple embryo transfer cycles. BMI, body mass index; PCOS, polycystic ovary syndrome.

**Supplementary Table S6. Comparisons of pregnancy outcomes of patients who underwent IVF/ICSI (n=812)**

|  | **≤30 years** | | **31~35 years** | | **≥36years** | |
| --- | --- | --- | --- | --- | --- | --- |
| **Characteristics** | PCOS  (n=41) | Control  (n=97) | PCOS  (n=56) | Control  (n=294) | PCOS  (n=20) | Control  (n=304) |
|  | Mean (SD)  or  n (%) | Mean (SD)  or  n (%) | Mean (SD)  or  n (%) | Mean (SD)  or  n (%) | Mean (SD)  or  n (%) | Mean (SD)  or  n (%) |
| **Age, years** | 28.5 (1.70) | 28.8 (1.49) | 32.7 (1.40) | 33.2 (1.35) | 37.8 (1.89) | 38.1 (1.65) |
| **BMI, kg/m^2^** | 27.8 (5.13) | 24.0 (4.98) | 25.1 (5.33) | 22.9 (3.80) | 24.4 (5.54) | 23.5 (4.55) |
| **Male infertility, Yes** | 36 (87.8%) | 92 (94.8%) | 52 (92.9%) | 271 (92.2%) | 17 (85.0%) | 271 (89.1%) |
| Normal–mild | 3 (7.3%) | 4 (4.1%) | 2 (3.6%) | 15 (5.1%) | 1 (5.0%) | 19 (6.3%) |
| Mild–moderate | 13 (31.7%) | 42 (43.3%) | 28 (50.0%) | 166 (56.5%) | 11 (55.0%) | 140 (46.1%) |
| Severe | 23 (56.1%) | 50 (51.5%) | 23 (41.1%) | 103 (35.0%) | 6 (30.0%) | 125 (41.1%) |
| **Total cycles** | 1.20 (0.464) | 1.28 (0.535) | 1.18 (0.431) | 1.37 (0.793) | 1.50 (1.00) | 1.59 (0.856) |
| **Number of oocytes retrieved** | 17.9 (10.6) | 14.8 (8.61) | 17.3 (7.87) | 14.4 (8.39) | 24.8 (15.3) | 12.6 (9.17) |
| **Average number of oocytes retrieved/cycle** | 14.7 (7.44) | 12.0 (6.89) | 15.0 (5.94) | 11.4 (6.33) | 16.0 (8.11) | 8.71 (6.55) |
| **Cumulative clinical pregnancy, Yes** | 26 (63.4%) | 59 (60.8%) | 36 (64.3%) | 174 (59.2%) | 14 (70.0%) | 84 (27.6%) |
| **Miscarriage, Yes** | 3/26 (11.5%) | 5/59 (8.5%) | 2/36 (5.6%) | 17/174 (9.8%) | 3/14 (21.4%) | 20/84 (23.8%) |
| **Live birth, Yes** | 21/26 (80.8%) | 53/59 (89.8%) | 29/36 (80.6%) | 136/174 (78.2%) | 9/14 (64.3%) | 61/84 (72.6%) |
| **Age at delivery** | 31.5 (2.07) | 31.4 (2.21) | 34.8 (1.80) | 35.1 (1.60) | 39.9 (1.45) | 39.5 (1.74) |

BMI, body mass index; PCOS, polycystic ovary syndrome.

**Supplementary Table S7: Assessment of oocyte quality in terms of usable blastocysts/oocyte retrieved and fertilized oocytes/oocyte retrieved in n=563 women who underwent IVF (1 stimulation cycle)**

|  | **PCOS** | **Control** | **Overall** | ***P*-value** |
| --- | --- | --- | --- | --- |
|  | **(n=95)** | **(n=468)** | **(n=563)** |  |
| **Useable blastocyst** |  |  |  |  |
| Mean (SD) | 4.35 (3.72) | 2.35 (3.06) | 2.66 (3.25) | <0.001 |
| Median (IQR) | 4.00 (7.00) | 0 (4.00) | 1.00 (5.00) |  |
| **Useable blastocyst/oocyte retrieved** |  |  |  |  |
| Mean (SD) | 0.271 (0.237) | 0.162 (0.191) | 0.179 (0.203) | 0.002 |
| Median (IQR) | 0.250 (0.395) | 0 (0.313) | 0.121 (0.333) |  |
| **Fertilized oocytes** |  |  |  |  |
| Mean (SD) | 8.77 (5.12) | 5.76 (4.09) | 6.23 (4.41) | <0.001 |
| Median (IQR) | 8.00 (7.00) | 5.00 (5.00) | 5.00 (6.00) |  |
| **Fertilized oocytes/oocyte retrieved** |  |  |  |  |
| Mean (SD) | 0.554 (0.226) | 0.496 (0.228) | 0.505 (0.228) | 0.11 |
| Median (IQR) | 0.571 (0.322) | 0.500 (0.333) | 0.500 (0.333) |  |
| **Fresh_D5** |  |  |  |  |
| Mean (SD) | 0.183 (0.500) | 0.171 (0.469) | 0.172 (0.474) | 0.788 |
| Median (IQR) | 0 (0) | 0 (0) | 0 (0) |  |
| **Fresh_D6** |  |  |  | NA |
| Mean (SD) | 0 (0) | 0 (0) | 0 (0) |  |
| Median (IQR) | 0 (0) | 0 (0) | 0 (0) |  |
| **Frozen_D5** |  |  |  |  |
| Mean (SD) | 3.00 (3.25) | 1.50 (2.46) | 1.74 (2.65) | <0.001 |
| Median (IQR) | 2.00 (6.00) | 0 (3.00) | 0 (3.00) |  |
| **Frozen_D6** |  |  |  |  |
| Mean (SD) | 1.17 (2.09) | 0.680 (1.33) | 0.758 (1.49) | 0.038 |
| Median (IQR) | 0 (2.00) | 0 (1.00) | 0 (1.00) |  |

D5, Day 5; D6, Day 6; PCOS, polycystic ovary syndrome.

**Supplementary Table S8: Assessment of E2:oocyte ratio in n=563 women who underwent IVF (1 stimulation cycle)**

|  | **≤30 years** | | **31~35 years** | | **≥36 years** | | **Overall** | |
| --- | --- | --- | --- | --- | --- | --- | --- | --- |
|  | **PCOS** | **Control** | **PCOS** | **Control** | **PCOS** | **Control** | **PCOS** | **Control** |
|  | **(n=33)** | **(n=74)** | **(n=47)** | **(n=213)** | **(n=15)** | **(n=181)** | **(n=95)** | **(n=468)** |
| **E2 pmol/L** |  |  |  |  |  |  |  |  |
| Mean (SD) | 21 600 (12100) | 16 400 (11800) | 19 700 (14000) | 13 100 (9060) | 20 500 (11700) | 14 900 (19100) | 20 400 (13000) | 14 300 (14 100) |
| Median (IQR) | 21 900 (14 600) | 13 900 (13 800) | 17 300 (21 200) | 11 500 (11 100) | 21 500 (19 500) | 10 400 (11 500) | 18 900 (19 900) | 11 300 (11 400) |
| **Number of oocytes retrieved** |  |  |  |  |  |  |  |  |
| Mean (SD) | 14.6 (7.98) | 13.2 (7.40) | 15.4 (6.18) | 12.5 (6.54) | 16.6 (9.30) | 10.1 (7.47) | 15.2 (7.16) | 11.6 (7.15) |
| Median (IQR) | 15.0 (13.0) | 11.5 (9.25) | 14.0 (6.75) | 11.5 (9.00) | 16.5 (17.0) | 9.00 (9.00) | 14.0 (10.0) | 10.0 (10.0) |
| **E2:number of oocytes** |  |  |  |  |  |  |  |  |
| Mean (SD) | 1640 (1210) | 1420 (879) | 1520 (1340) | 1260 (1090) | 1550 (1220) | 1780 (2010) | 1560 (1270) | 1480 (1500) |
| Median (IQR) | 1300 (1120) | 1120 (994) | 1210 (1140) | 1010 (1110) | 1360 (574) | 1420 (1420) | 1250 (1110) | 1120 (1120) |

E2, estradiol; PCOS, polycystic ovary syndrome.

**Supplementary Table S9: Assessment of E2/oocyte ratio in n=563 women, with ethnicity breakdown, who underwent IVF (one stimulation cycle)**

|  | **Chinese** | **Malay** | **Indian** | **Others** | ***P*-value** |
| --- | --- | --- | --- | --- | --- |
|  | **(n=357)** | **(n=76)** | **(n=64)** | **(n=66)** |  |
| **E2 pmol/L** |  |  |  |  |  |
| Mean (SD) | 15 000 (15700) | 19 200 (11700) | 13 100 (9980) | 14 600 (8850) | 0.002 |
| Median (IQR) | 11 700 (11 600) | 17 600 (19 600) | 9270 (13 900) | 14 000 (9690) |  |
| **Number of oocytes retrieved** |  |  |  |  |  |
| Mean (SD) | 12.6 (7.42) | 12.5 (6.16) | 11.8 (7.65) | 10.4 (7.14) | 0.079 |
| Median (IQR) | 12.0 (10.0) | 11.0 (8.00) | 10.0 (8.50) | 9.00 (8.00) |  |
| **E2/number of oocytes** |  |  |  |  |  |
| Mean (SD) | 1450 (1650) | 1610 (1000) | 1370 (1120) | 1730 (1030) | 0.003 |
| Median (IQR) | 1060 (1140) | 1280 (1280) | 1110 (1040) | 1490 (1200) |  |

E2, estradiol.
